# Rare cases in two Chinese MEN2A families with RET C634Y germline mutation—a homozygous female patient and heterozygous identical twins: a systematic review of literature

**DOI:** 10.3389/fendo.2026.1690431

**Published:** 2026-02-06

**Authors:** Xiao-Ping Qi, Yu-Ting Weng, Zhen-Yu Chen, Mei-Xian Zhang, Xiao-Ling Yang, Zhi-Lie Cao, Xue-Xing Zhang, Wen-Bo Zhu, Hong-Yuan Yu, Wei-Ying Chen, Ji-Ya Chen, Fei-Ping Li

**Affiliations:** 1 Department of Urology, Taizhou Hospital of Zhejiang Province Affiliated to Wenzhou Medical University, Enze Hospital of Hangzhou Medical College, Taizhou Enze Medical Center (Group), Taizhou, China; 2 Department of Oncologic and Urologic Surgery, The 903rd People's Liberation Army (PLA) Hospital, Hangzhou Medical College, Hangzhou, Zhejiang, China; 3 Evidence-Based Medicine Center, Taizhou Hospital of Zhejiang Province Affiliated to Wenzhou Medical University, Enze Hospital of Hangzhou Medical College, Taizhou Enze Medical Center (Group), Taizhou, China; 4 Wenzhou Medical University, Wenzhou, Zhejiang, China; 5 Department of Thyroid and Breast Surgery, Taizhou Enze Medical Center (Group) Enze Hospital, Taizhou, Zhejiang, China; 6 Taizhou Hospital of Zhejiang Province Affiliated to Wenzhou Medical University, Enze Hospital of Hangzhou Medical College, Taizhou Enze Medical Center (Group), Taizhou, China

**Keywords:** homozygosity, medullary thyroid carcinoma, monozygotic twins, multiple endocrine neoplasia type 2, pheochromocytoma, RET proto-oncogene

## Abstract

**Background:**

Germline *RET-*p.C634Y heterozygous mutations are predominant in MEN2A, but homozygous cases and MEN2A-affected identical twins remain poorly characterized.

**Summary:**

We report two MEN2A families—a homozygous female patient and heterozygous male twins, all with *RET-*p.C634Y mutations and classic MEN2A manifestations. A systematic review identified 18 homozygous cases from 10 families, involving exons 11, 14, and 15, containing nine types of mutations, presenting a female (55.6%) and moderate-risk mutation (61.1%) predominance. Overall, 83.3% of the 18 patients with homozygous mutations and 30.6% of the 49 patients with heterozygous mutations from the same generation had medullary thyroid carcinoma (MTC). The homozygous mutations had a higher penetrance rate of MTC (*P* < 0.001) and rates of node-positive metastasis (8/15 vs. 1/15, *P* = 0.017). However, the comparison of the mean age at initial MTC diagnosis between patients with homozygous and heterozygous mutations [33.40 ± 17.97 (5–59) vs. 39.60 ± 12.94 (14–61) years], as well as in moderate-risk and high-risk patients with homozygous mutations [36.89 ± 16.21 (13–59) vs. 28.17 ± 20.72 (5–56) years], showed no significant differences (all *P* > 0.05). Additionally, the mean age at diagnosis and the incidence of pheochromocytoma did not differ significantly [(37.75 ± 18.43) vs. (39.5 ± 3.54); 27.8% vs. 13.3%; all *P* > 0.05]. Clustered data for identical twins diagnosed with MEN2 were also analyzed, including one with MEN2A and two with MEN2B. All three pairs of identical twins exhibited varying clinical presentations, expressivity of MEN2-related MTC and/or pheochromocytoma, and associated biomarker levels.

**Conclusions:**

Homozygous MEN2A accelerates MTC onset and increases metastasis risk, but there is no evidence of association with the development of pheochromocytoma. Consanguineous marriage could increase homozygosity in offspring and the number of affected individuals. Expressivity and clinical progression can vary even with the same genetic backgrounds, and identical twins should also be subject to individual management.

## Introduction

Multiple endocrine neoplasia type 2 (MEN2) is a rare neuroendocrine tumor syndrome that follows an autosomal dominant inheritance pattern. It is characterized by medullary thyroid carcinoma (MTC), which may or may not be accompanied by pheochromocytoma (PHEO), hyperparathyroidism (HPTH), and extra-endocrine features (1, 2). Clinically, there are two distinct subtypes: MEN2A (OMIM# 171400; approximately 95% of MEN2 cases) and MEN2B (OMIM# 162300; approximately 5%) (1). Nearly all cases of MEN2 are caused by gain-of-function germline mutations in the REarranged during Transfection (*RET*) proto-oncogene (OMIM# 164761) (1, 2). Over the past three decades, new insights into the natural progression of the disease and genotype–phenotype correlations have led to a paradigm shift in the management of MEN2. This has resulted in significant advancements in prevention, prediction, individualized precision therapy, and comprehensive treatment, effectively promoting the transition from symptom-based to preventive healthcare (1–7). However, the diversity of mutant forms and tumor heterogeneity within the known genotype–phenotype relationships still need further clarification to enhance early diagnosis and individualized treatment, thereby improving efficacy (1–4).

To date, most *RET* mutations in MEN2 have been heterozygous missense mutations, although double/multiple mutations, insertion/deletion mutations, duplication mutations, or homozygous mutations also occur (https://www.ncbi.nlm.nih.gov/clinvar/). Instances of homozygous *RET* mutations are, however, extremely rare (1, 8–16). Herein, we report on a female patient with a homozygous *RET*-p.C634Y germline mutation who developed bilateral MTC and bilateral PHEO. Additionally, we conducted a systematic review of the literature to analyze the clinical data of other families with homozygous *RET* germline mutations reported previously, aiming to elucidate the presentation characteristics of MEN2 with homozygous mutations compared to those with heterozygous mutations of *RET*. Furthermore, we identified a pair of identical twin male patients with the *RET*-p.C634Y germline mutation, both of whom presented with bilateral MTC and unilateral PHEO. Upon meticulously examining patients’ medical histories, gathering biochemical and imaging data, and integrating literature reviews (17–20), we compared the expressivity and disparate disease progressions under identical genetic backgrounds.

## Materials and methods

### Study population

**Figure 1:**
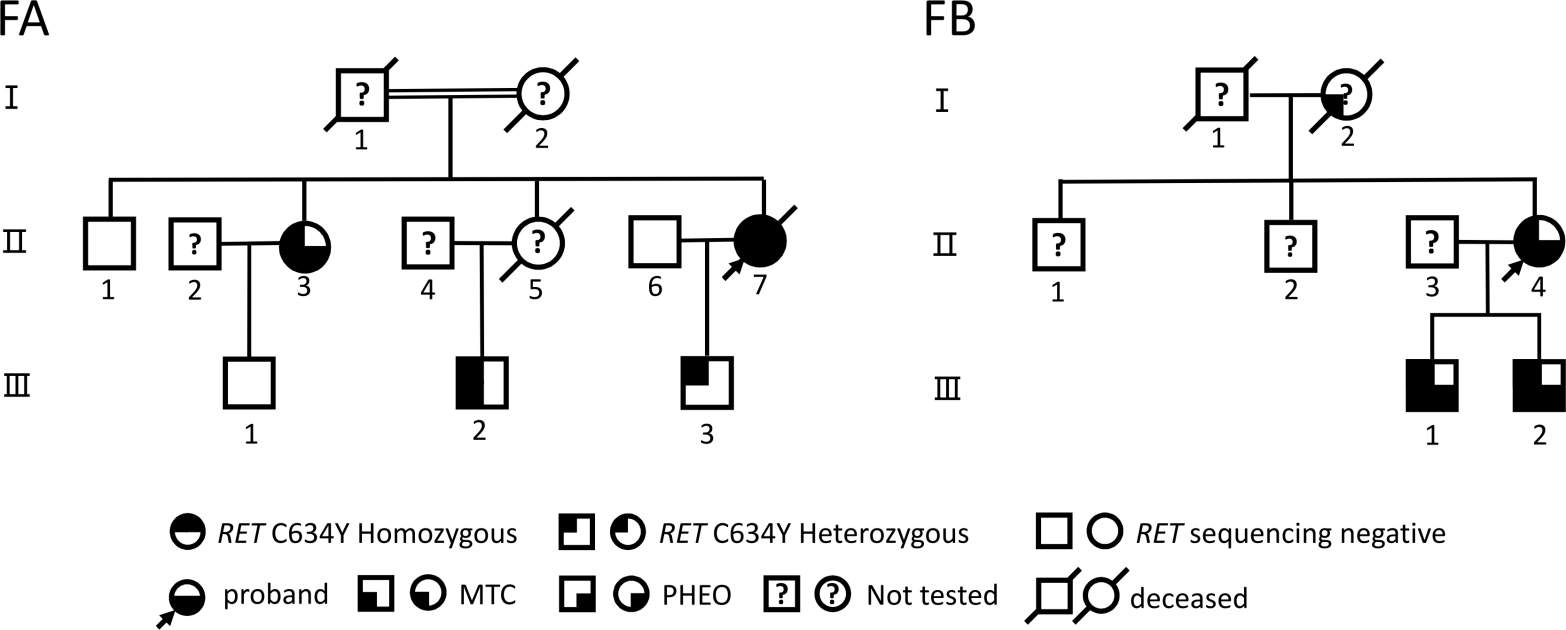
Genealogies of two MEN2A families with the *RET* C634Y mutation. Numbers indicate family members. Circles and squares indicate female (F) and male (M) patients, respectively. MTC, medullary thyroid carcinoma; PHEO, pheochromocytoma; HPTH, hyperparathyroidism.

These two pedigrees (**Figure 1**, **Table 1**), involving seven individuals diagnosed and treated for MEN2A at the 903rd PLA Hospital and Taizhou Enze Medical Center (Group) (Zhejiang, China) from June 2019 to August 2023, were selected for investigation using pedigree analysis and clinical and biochemical/imaging examinations, as described previously (21, 22): serum basal calcitonin [Ctn; normal < 8.4 pg/mL (male patients) and <5.0 pg/mL (female patients)], carcinoembryonic antigen (CEA; normal < 5.0 ng/mL), parathyroid hormone (PTH; 15–65 pg/mL), serum calcium, and plasma excreted amounts of catecholamines, metanephrines (MN; normal < 62 ng/L), and normetanephrines (NMN; normal < 145 ng/L). Imaging examinations involved Doppler B-ultrasound (US), computed tomography (CT), magnetic resonance imaging (MRI), and/or positron emission tomography-CT (PET-CT). According to the revised American Thyroid Association (ATA) Guidelines for the management of MTC, patients carrying the *RET* mutation are in the “moderate risk” (ATA-MOD), “high risk” (ATA-H), and “highest risk” (ATA-HST) categories (1). Postoperative tumor staging was performed using the American Joint Committee on Cancer version 8 TNM (tumor–node–metastasis) classification system (23). The subjects were followed up until November 2024.

**Table 1:** Clinical characteristics of *RET* C634Y mutation carriers in the two Chinese MEN2A pedigrees with a homozygous female patient and heterozygous identical twins.

Family/individual	*RET* mutation	Sex (M/F)	Age (years)	Age at diagnosis (years)			Pre-/post- Ctn (pg/mL)	MTC			PHEO		Time of follow-up (months)
				MTC	PHEO	HPTH		Surgery extension	Max size (L/R, cm)	TNM	Surgery extension	Size (L/R, cm)	
FAII-3	C634Y/wt	F	51	46	42	−	879/85.4	TT + BiLND(VI) + MLND	0.8/1.9	T1bN1bM0	ASS (Bi)	6.0/2.5	116
FAII-7 (proband)	C634Y/C634Y	F	42 (died)	25^①^ 39^②^	39	−	NA/>2,000	RT + ST (L)^①^	1.6/3.0^①^; 4.2/NA^②^	cT2NxMx^①^ cT3N1bMx^②^	Rejected	2.5/6.6	202
FAIII-2	C634Y/wt	M	22	22	WWW	−	32.78/<2	TT + BiLND(VI)	0.3/0.3	T1aN0M0	WW	WW	9
FAIII-3	C634Y/wt	M	21	21	−	−	3.01/NA	WW	NA	NA	−	−	9
FBII-4 (proband)	C634Y/wt	F	51	21	−	43	NA/34.97	ST (L)^①^ + RT^②^	NA	NA	−	−	360
FBIII-1 ^#^	C634Y/wt	M	23	16	18, 21	−	436.6/2.87	TT + BiLND(VI)^①^ + MRND^②^	0.6/1.0	T1aN1aMx^①^; T1aN1bM0^②^	ASS (L)	4.8/1.0	88
FBIII-2 ^#^	C634Y/wt	M	23	16	22	−	>2,000/73.3	TT + BiLND(VI) + MLND^①^ + MRND^②^	2.5/0.5	T2N1bMx^①^; T2N1bM0^②^	ASS (L)	6.5/−	88

MEN2A, multiple endocrine neoplasia type 2A; M/F, male/female; MTC, medullary thyroid carcinoma; PHEO, pheochromocytoma; HPTH, hyperparathyroidism; Pre-/post-Ctn, pre-/post-surgery basal serum calcitonin (normal for men and women is <8.4 and <5.0 pg/mL, respectively); FA, family A; FB, family B; Max size, maximum MTC size (all initial); L/R, left or right; Bi, bilateral; TT, total thyroidectomy; BiLND(VI), bilateral level VI lymph node dissection; M (L/R)ND, modiﬁed (left/right) neck dissection; RT, right thyroidectomy; ST, subtotal thyroidectomy; MBiND, modiﬁed bilateral neck dissection; TNM, tumor–node–metastasis (tumor stage); ASS, adrenal-sparing surgery; WW, watchful waiting; NA, not available.

−, negative; ^#^, twins.

① Initial surgery.

② Second surgery.

### Genetic testing and haplotype analysis

Briefly, genomic DNA was isolated from the proband and family members, followed by targeted sequencing with the Target Cap pheochromocytoma/paraganglioma Panel^®^ (CRED BIO, Hangzhou, China). Quality was assessed using the Bioptic Qsep100 and ABI StepOne, and sequencing was performed on the MGI DNBSEQ-T7 (MGI, Wuhan, China). Pathogenic mutations were identified using the Human Gene Mutation Database and ClinVar and validated by Sanger sequencing in accordance with the American College of Medical Genetics and Genomics guidelines.

For haplotype analysis, DNA from the twins was extracted using the HiPure Blood DNA Mini Kit (Magen, Guangzhou, China. Polymerase chain reaction amplification was performed with the Quick-insight A22 Human DNA Identification Kit (XY Biotechnology, Hangzhou, China), assessed using the SHSST electrophoresis system (SHST, Hangzhou, China), and sequenced on the ABI 3730XL (Life Tech, California, USA).

### Literature search strategy and data collection

Two authors (YTW, WYC) independently searched the Cochrane Library, PubMed, Web of Science, Scopus, and Embase up to October 2024 using the following MeSH and Title/Abstract terms: #1 (“homozygous” OR “homozygosity”); #2 (“medullary thyroid cancer” OR “medullary thyroid carcinoma”); #3 (“pheochromocytoma” OR “phaeochromocytoma”); #4 (“hyperparathyroidism” OR “parathyroid adenoma” OR “parathyroid tumor”); and #5 (#1 AND [#2 OR #3 OR #4]). Additionally, an electronic search using the following MeSH and Title/Abstract terms was also conducted: (“multiple endocrine neoplasia type 2” OR “multiple endocrine neoplasia type II” OR “MEN 2” OR “MEN II” OR #2 OR #3) AND “twin.” No language or time restrictions were applied. This search respectively retrieved 775 publications. The full texts of the articles were evaluated for eligibility. Any discrepancies were discussed and rechecked, verified by a third reviewer (XPQ) (**Supplementary Figure 1**). A total of eight (8–15) and two (19, 20) publications were respectively identified for “homozygous *RET* germline mutations” or “twins” (as presented in **Tables 2**, **3**).

**Table 2:** MEN2A phenotype in reported *RET* homozygotes and in our present study.

References	Homozygous mutation (no. of families)	Nucleotide change	No. of carriers	Sex (M/F)	MTC				PHEO			HPTH
					No. of patients (%)	AD (years)	MTD (cm)	Proportion of node-positive	No. of patients (%)	AD (years)	MTD (cm)	No. of patients (%)
Demir et al. (14)	S891A^*^ (1)	c.2671T > G	1	1/0	1 (100)	15	2	1/1	0 (0)	−	−	−
Elisei et al. (10)	A883T^#^ (1)	c.2647G > A	2	2/0	2 (100)	51, 56	0.3	0/2	0 (0)	−	−	−
Lecube et al. (9) Lesueur et al. (11)	V804M (2)	c.2410G >A	4	3/1	2 (50)^§^	13, 30	0.3	1/2	0 (0)	−	−	−
			2	0/2	2 (100)	32, 54	−	0/2	0 (0)	−	−	−
Lesueur et al. (11)	V804L (1)	c.2410G > C	1	0/1	1 (100)	37	−	1/1	1 (100)	39	−	−
Lesueur et al. (11)	V804L (1)	c.2410G > C	1	0/1	1 (100)	37	−	1/1	1 (100)	39	−	−
Vuylsteke et al. (15)	c.1998delinsTTCT (1)	c.1998delinsTTCT	2	1/1	2 (100)	42, 50	−	2/2	0(0)	−	−	−
Jaber et al. (12)	K666N (1)	c.1998G > C (T)	1	0/1	1 (100)	59	1.6	1/1	1 (100)	58	5	−
Huang et al. (8)	C634F (1)	c.1901G > T	2	0/2	1 (50)	18	−	0/1	0(0)	−	−	−
Schirwani et al. (13)	C634W (1)	c.1902C > G	2	1/1	2 (100)	5, 14	4.2;0.9	1/2	1 (50)	14	−	−
Present study	C634Y (1)	c.1901G > A	1	0/1	1 (100)	25	5	1/1	1 (100)	39	6.55	−
**Total** ^a^	10^b^		**18** ^c^	**8/10**	**15 (83.3)**	33.4^d^	**2.04** ^e^	**8/15**	**5 (27.7)**	37.5^f^	**5.78** ^g^	−

M, male; F, female; MTC, medullary thyroid carcinoma; PHEO, pheochromocytoma; HPTH, hyperparathyroidism; AD, age at diagnosis; MTD, maximum tumor diameter; −, negative; NID, not identified to date.

* The patient with both *NF1* (c.5610-2A>G) and homozygous *RET* (c.2671T>G) germline mutations.

# Compared to the other four heterozygous mutation patients, who lacked the MTC phenotype and MEN2-related symptoms.

§ Of the four carriers, one (15 years) had MTC with lymph nodes of the neck and muscle, one (30 years) presented C-cell hyperplasia, and the remaining two (5 and 10 years, respectively) had no abnormality of thyroid and calcitonin levels.

a Summarizes data from previously published literature (excluding the present study).

b Total no. of families

c Total no. of carriers.

d The mean age at the time of MTC diagnosis in 15 of all MEN2A patients was 33.4 years.

e The mean maximum tumor diameter of MTC is 2.04cm.

f The mean age at the time of PHEO diagnosis in the 4 patients was 37.5 years.

g The mean maximum tumor diameter of PHEO is 5.78cm.

**Table 3:** Presentation of expressivity (heterogeneity) of MEN2 in twins.

References	*RET* mutation/MEN2	Sex (M/F)	Presentation at the time of initial diagnosis	MTC^§^					PHEO^§^			
				Age at diagnosis (years)	Pre-Ctn (pg/mL)	Pre-CEA (ng/mL)	Max size (L/R, cm)	TNM stage	Age at diagnosis (years)	CA (ng/L)	MN/NMN (ng/L)	Max size (L/R, cm)
Reza et al. (19)	NA/MEN2B^C^	M	Enlarged thyroid nodule, peculiar facies, high-arched palate, tongue neuromas, pes cavus	18	NA	NA	“Enlarged”	TxNxMx	19	+	NA	“36 g”/−
		M		18	NA	NA	“Enlarged”	TxN1Mx	21	+	NA	+/”18 g”
Galera et al. (20)	NA/MEN2B^C^	M	Diarrhea, palpitations, hot flushing, dark skin, Marfan-like aspect, thick lips, tongue nodular, and scality genital development	14	72,000	NA	0.5~1.2	T1N1bM0^#^	−	−	NA	−
		M		14	65,000	NA	0.5~1.2	T1N1bM1^#^ (left lung metastatic)	−	−	NA	−
Present study	C634Y/MEN2A	F	Asymptomatic	16	431.2	13.2	0.6/1.0	T1aN1aM0	18	+	2,502/1,980	4.8/−
		F	Neck mass	16	>2,000	44.1	2.5/0.5	T2N1bM0	22	+	271/169	6.5/−

MEN2A, multiple endocrine neoplasia type 2A; MTC, medullary thyroid carcinoma; PHEO, pheochromocytoma; Pre-Ctn, pre-surgery basal calcitonin; L/R, left or right; TNM, tumor–node–metastasis (tumor stage); CA, catecholamines (epinephrine, norepinephrine, and dopamine); MN, metanephrine; NMN, normetanephrine; −, negative; +, positive.

C Clinical diagnosis.

§ At the time of initial diagnosis.

# Suspiciously.

### Statistical analysis

To conduct the statistical evaluation, the software package SPSS^®^ version 28 (IBM, Armonk, NY, USA) was utilized. Continuous variables are summarized as mean ± standard deviation (mean ± SD). The Shapiro–Wilk test was employed to assess whether the data followed a normal distribution. For normally distributed continuous variables, the independent samples *t*-test was used to compare the mean differences between two groups. In the case of a small sample size, the Mann–Whitney *U* test (*U* test) was used for group comparison. For the statistical comparison of binary data, Fisher’s exact test was utilized to assess the significance of differences between groups. All *P*-values <0.05 were considered to indicate statistical significance.

## Results

### Clinical features and phenotypic data

#### Family A

**Figure 2:**
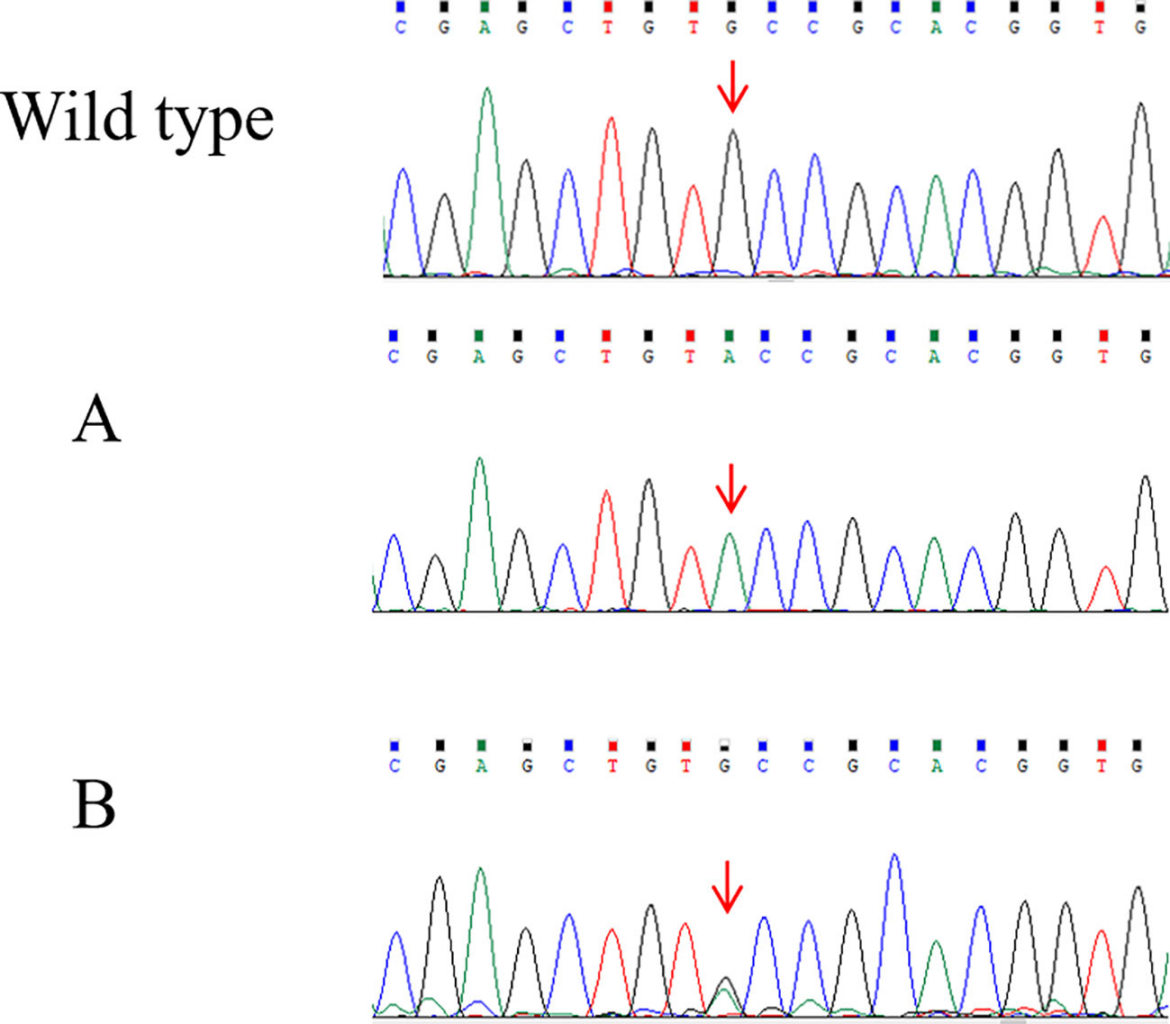
DNA sequencing confirmed the *RET* mutations in the two families. **(A)** A homozygous missense mutation of p.C634Y (c.1901G>A) in exon 11 of the *RET* gene in the proband (FAII-6) of family A. **(B)** A heterozygous missense mutation of p.C634Y (c.1901G>A) in exon 11 of the *RET* gene in the twin sons (FBIII-1) of family B.

The proband (FAII-7; **Figure 1**), a 25-year-old woman, was admitted to a local hospital in August 2006 with a 6-year history of a neck mass (**Table 1**). US examination indicated bilateral thyroid multi-hypoechoic nodules (left, 1.6 cm; right, 3.0 cm) and a suspicion of a diagnosis of “bilateral thyroid adenoma.” She then underwent a right thyroidectomy and a left subtotal thyroidectomy. Pathological examination revealed bilateral “thyroid adenomas.” In August 2020, she returned to a local hospital due to a recurrent palpable neck mass. Both US and CT revealed a 4.2-cm hypoechoic nodule with microcalcifications in the left thyroid lobe, along with cervical lymphadenopathy. Her Ctn and CEA levels were >2,000 pg/mL and 147.9 ng/mL, respectively, suggesting left MTC with cervical lymph node metastases. Subsequently, she visited our hospital. Abdominal US and CT scanning revealed bilateral adrenal masses (left, 2.5 cm; right, 6.6 cm). Her MN and NMN levels were 137 nmol/L and 629 nmol/L, respectively. *RET* testing revealed that the proband carried a homozygous p.C634Y (c.1901G>A) germline mutation (**Figure 2A**), while re-evaluation of the initial thyroid pathology slides suggested bilateral MTC with focal invasion of the thyroid capsule and vascular invasion. The final diagnosis was “classical MEN2A-related MTC (cT3N1bMx) and bilateral PHEO.” Unfortunately, her family opted for conservative symptomatic treatment rather than undergoing further appropriate surgical treatment. The patient passed away at home in June 2023 due to multiple injuries from an accident.

In March 2015, the proband’s 42-year-old sister (FAII-3) underwent bilateral adrenal-sparing surgery due to bilateral adrenal masses (left, 6.0 cm; right, 2.5 cm) and hypertension (blood pressure ranging from 95 to 190/55 to 110 mmHg). Pathological results confirmed bilateral PHEO. In August 2019, she underwent a total thyroidectomy with bilateral level VI lymph node dissection and modified right neck dissection due to bilateral hypoechoic thyroid nodules (left, 1.0 cm; right, 2.2 cm) identified by both US and CT examinations, along with elevated levels of CEA at 60.5 ng/mL and Ctn at 879 pg/mL. Histopathological examination revealed bilateral MTC with lymph node metastases (LN+/resected, 11/32; T1bN1bM0). In June 2022, both US and CT scans revealed a residual right thyroid lobe measuring 1.6 cm × 0.6 cm and bilateral adrenal masses (left, 2.3 cm × 1.4 cm; right, 1.3 cm × 1.1 cm), with Ctn, MN, and NMN levels elevated to 85.4 pg/mL, 16.1 nmol/L, and 385 nmol/L, respectively, suggesting a recurrence of MTC and bilateral PHEO. However, the patient declined further treatment and opted for a watchful waiting approach.

In February 2024, initially hesitant family members consented to participate in a family screening (**Figure 1**). Out of the six members, three displayed normal US images and Ctn levels, while the other three (FAII-3, FAIII-2, and FAIII-3; see **Table 1**) were found to have the heterozygous *RET-*p.C634Y mutation (c.1901G>A) (**Figure 2B**). Subsequently, the proband’s nephew (FAIII-2) underwent a total thyroidectomy with bilateral level VI lymph node dissection. Histopathological findings indicated bilateral MTC (LN−/resected, 0/15; T1aN0M0); postoperative Ctn levels decreased from 32.78 to undetectable (<2 pg/mL), whereas his MN levels remained slightly elevated at 79.2–110.7 ng/L. CT scanning revealed a tiny right adrenal nodule (0.3~0.5 cm) with microcalcification. The proband’s son (FAIII-3) exhibited a left thyroid nodule (0.4 cm) and marginally elevated CEA levels (8.9~9.3 ng/mL), yet his Ctn levels (3.01~4.62 pg/mL) were within the normal range, and there was no evidence of PHEO. The diagnosis is “suspicion of MEN2-related MTC or C-cell hyperplasia.” He was hesitant to undergo a prophylactic thyroidectomy and had opted for watchful waiting.

Additionally, the proband’s parents (FAI-1 and FAI-2), who were consanguineously married, likely died of myocardial infarction at age 46 or died of uremia at age 59, respectively. Her older sister (FAII-5) passed away unexpectedly at age 27 due to “high blood pressure (170/120 mmHg) and myocardial infarction” following a cold.

#### Family B

The proband (FBII-4; **Figure 1**), a woman, underwent a left subtotal thyroidectomy at age 21 in 1994 and a right total thyroidectomy at age 26 in 1999 due to palpable neck masses at a local hospital. Histopathology confirmed MTC (**Table 1**). In October 2014, at the age of 43, she experienced sudden limb convulsions during sleep one night. Biochemical examinations showed elevated levels of Ctn (28.61 pg/mL) and PTH (139.9 pg/mL) and decreased 25-hydroxyvitamin D (24.78 nmol/L; reference range > 50 nmol/L), although serum calcium levels remained within normal limits. After beginning daily oral vitamin D supplementation of 800 IU, her symptoms improved. During hospitalization, a 2.4-cm × 0.7-cm nodule in the lower neck and a PTH suppression rate of 73% by high-calcium suppression test were detected, strongly suggesting parathyroid adenoma (HPTH). The endocrinologist recommended that surgery for parathyroid adenoma could be deferred, while the presence of PHEO had initially been ruled out by biochemical and CT examinations. Family history revealed that her mother had died of unknown causes at age 80, had undergone MTC surgery at age 30, and had a history of osteoporosis for many years. In September 2016, her Ctn, CEA, and PTH levels fluctuated between 28.61 and 34.97 pg/mL, 4.79 and 6.02 ng/mL, and 139.9 and 186.61 pg/mL, respectively. She then underwent a US-guided FNA on the lower neck nodule, and cytological examination suggested a possible “parathyroid adenoma.” Genetic testing revealed that the proband and her twin sons (FBIII-1 and FBIII-2) carried a heterozygous *RET*-p.C634Y mutation (**Figure 2B**).

In July 2017, her 16-year-old twin sons exhibited biochemical abnormalities or a neck mass upon imaging examinations. Specifically, the older twin (FBIII-1) had bilateral thyroid nodules (left, 0.6 cm; right, 1.0 cm) and elevated levels of Ctn (431.2 pg/mL) and CEA (13.2 ng/mL). The younger twin (FBIII-2) presented with bilateral thyroid nodules (left, 2.5 cm; right, 0.5 cm) and significantly increased Ctn (>2,000 pg/mL) and CEA (44.1 ng/mL) levels (**Figure 1**, **Table 1**). Subsequently, they underwent total thyroidectomies with bilateral level VI lymph node dissection (FBIII-1) and/or modified left neck dissection (FBIII-2) at another hospital, respectively. Histopathological examination revealed bilateral MTC with lymph node metastasis (FBIII-1, LN+/resected, 1/4; T1aN1aM0; FBIII-2, LN+/resected, 2/13; T2N1bM0).

In June 2019, the older twin (FBIII-1) experienced occasional paroxysmal headaches. His blood pressure ranged from 160 to 110/95 to 60 mmHg. Both US and CT scans revealed a 4.8-cm × 4.0-cm mass in the left adrenal gland. Plasma levels of MN and NMN were elevated to 2,502 and 1,980 ng/L, respectively. After a week of treatment with terazosin hydrochloride and volume expansion, he underwent laparoscopic left adrenal-sparing surgery. The pathological report indicated a left PHEO. In August 2022, several hypoechoic nodules were detected in the right neck with an elevated Ctn level of 188 pg/mL and a CEA level of 10.14 ng/mL. Additionally, a mass approximately 1.0 cm in diameter was found in the right adrenal gland, but there were no symptoms, and the levels of NMN and MN were within normal limits. FNA was performed on the neck nodules, and the results suggested the possibility of metastatic MTC. Consequently, he underwent right lateral neck lymph node dissection, and histopathological examination confirmed right-sided MTC (LN+/resected, 8/50; T1aN1bM0). Postoperative levels of Ctn and CEA have gradually declined to 2.87 and 5.03 ng/mL, respectively (**Table 1**).

In July 2023, the younger twin (FBIII-2) was found to have a 6.5-cm × 3.0-cm nodule in the left adrenal gland, with elevated levels of MN and NMN (271 ng/L and 169 ng/L, respectively). Subsequently, laparoscopic left adrenal-sparing surgery was performed after a week of treatment with terazosin hydrochloride. Pathological results confirmed a left PHEO. In August 2023, both US and CT examinations revealed several hypoechoic nodules in the right neck, with Ctn levels elevated to 209 pg/mL and CEA at 8.01 ng/mL. FNA results suggested the possibility of MTC. He underwent a modified right neck lymph node dissection, and the pathology report indicated metastatic MTC (LN+/resected, 5/40; T2N1bM0). His Ctn and CEA levels dropped to 73.3 and 5.05 ng/mL, respectively (**Table 1**).

In March 2024, further haplotype analysis of 22 *RET*-flanking microsatellite markers, whose chromosome map distances were derived from the deCODE map (https://www.ncbi.nlm.nih.gov/probe/), was performed to confirm that the twins’ genes are identical. The results, which were consistent, are presented in **Supplementary Table S1**. However, their mother (proband) declined further thorough examination and treatment.

### Clustered data for patients with homozygous germline RET mutations

Combining our series with literature reports, the clinical presentation of 18 homozygous and 15 out of 49 heterozygous patients from the same generation within the corresponding 10 families is detailed in **Table 2**. There was no significant difference in gender distribution between homozygous and heterozygous patients (*P* > 0.05). Among the specific 18 homozygous patients, 16 with missense mutations (88.9%) and 2 with insertion–deletion mutations (11.1%) were identified. The two most common mutations were p.V804M/L (38.9%, 7/18) and p.C634F/W/Y (27.8%, 5/18). Classification into ATA-H was observed in 5 cases (27.8%; p.C634F/W/Y), while ATA-MOD was present in 13 cases (72.2%; p.V804M/L, K666N, A883T, S891A, and c.1998delinsTTCT). No patient carried the MEN2B-harboring ATA-HST homozygous *RET* mutation.

In all, 83.3% of 18 patients with homozygous and 30.6% of 49 patients with heterozygous mutations had MTC (15/18 vs. 15/49; *P* < 0.001), while the comparison of the rate of MTC node positivity (N1 metastasis) between patients with homozygous and heterozygous mutations [53.3% (8/15) vs. 6.7% (1/15); *P* = 0.017] revealed a significant difference, with homozygous patients having a higher penetrance rate of MTC and exhibiting higher rates of invasiveness or metastasis than those with heterozygous mutations (**Supplementary Table S2**). The comparison of the mean age at initial MTC diagnosis showed no significant difference between patients with homozygous and heterozygous *RET* mutations [33.40 ± 17.97 (5–59) vs. 39.60 ± 12.94 (14–61) years; *P* = 0.144]. Similarly, when stratified by ATA risk categories, the mean diagnostic ages of ATA-MOD and ATA-H subgroups did not differ significantly between homozygous and heterozygous patients [ATA-MOD: 36.89 ± 16.21 (13–59) vs. 44.33 ± 11.17 (30–61) years, *P* = 0.137; ATA-H: 28.17 ± 20.72 (5–56) vs. 32.50 ± 12.99 (14–46) years, *P* = 0.337] (**Supplementary Table S3**).

Regarding PHEO, 27.8% of homozygous and 13.3% of heterozygous patients were affected (5/18 vs. 2/15; *P* = 0.067). The mean age at initial PHEO diagnosis was also comparable between the two groups [37.75 ± 18.43 (14–59) vs. 39.5 ± 3.54 (37–42) years; *P* = 1.000, Mann–Whitney *U* test] (**Supplementary Table S2**). Although the youngest homozygous patient was 23 years younger than the youngest heterozygous case, the overall difference was not statistically significant.

### Clustered data for twin patients with MEN2

Two publications (two MEN2A) were excluded due to the unavailability of full text or detailed clinical information (20, 21). This left a total of two reports and our own series for analysis, including one with MEN2A and two with MEN2B. All three pairs of identical twins exhibited varying clinical presentations (symptomatic or asymptomatic), presented differential expressivity and tumor-cell heterogeneity of MEN2-related MTC and/or PHEO (age of onset, tumor size, weight, and staging), and had different degrees of biochemical levels (Ctn, CEA, catecholamines/MNs) (see **Table 3**). These findings indicate that, beyond genetic abnormalities in the *RET* gene, there may be additional factors influencing the occurrence and development of tumors in MEN2 syndrome.

## Discussion

Homozygous *RET* mutations in MEN2A are extremely rare. Although *RET*-p.C634 mutations are the predominant pathogenic variants worldwide, most reported cases occur in the heterozygous state, and true homozygous individuals have only been described in isolated families. These homozygous mutations differ from the inactivating *RET* variants reported in Hirschsprung disease, which are associated with developmental defects rather than oncogenic activation (24, 25).

This study presents the first report of a case involving a female patient (FAII-7) with a homozygous *RET*-p.C634Y mutation (c.1901G>A) who developed bilateral MTC and PHEO, with her parents being consanguineous (**Table 1**). The patient exhibited noticeable thyroid nodules at age 19, and by age 25, the thyroid was compressing the trachea, with a Ctn level >2,000 pg/mL (T3N1bMx). In contrast, her older sister (FAII-3), who carries a heterozygous mutation, first detected thyroid nodules at age 42, which became palpable by age 46, without tracheal compression and with a pre-Ctn level of 879 pg/mL (T1bN1bM0). The findings suggest that MTC in homozygous individuals appears to occur earlier, progress more rapidly, and have a higher TNM staging. However, there appears to be no difference in the occurrence and progression of PHEO. The cause of death for her younger sister (FAII-5, aged 27) as “high blood pressure and myocardial infarction” raises questions about whether she was a homozygous carrier or had MEN2A-related PHEO; the exact cause remains undetermined. Regrettably, her son (FAIII-3) had been diagnosed with “suspicion of MEN2-related MTC or C-cell hyperplasia,” yet he had thus far declined prophylactic thyroidectomy and had chosen active surveillance.

After the systematic literature review, we collected the clinical characteristics of 18 cases with MEN2A and homozygous mutations between November 2004 and October 2024. A female (55.6%) and ATA-MOD mutation (61.1%) predominance was shown, and there were no patients with MEN2B and homozygous mutations (**Table 2**). Fifteen out of 18 (83.3%) developed MTC.

The mean initial diagnostic age was 33.4 years (range, 5–59) in homozygous patients, approximately 6.2 years younger than that in heterozygous patients [39.6 years (range, 14–61)], but the difference was not statistically significant (*P* = 0.144). On the other hand, homozygous patients showed a diagnostic age that was on average 9 years younger and a significantly higher MTC penetrance rate (*P* < 0.001) compared with heterozygous patients. Excluding three cases without abnormality, 53.3% of 15 homozygous patients with MTC had N1 metastasis, with significantly higher rates of invasiveness, compared to only 6.7% of 15 heterozygous patients (*P* = 0.017), indicating a higher frequency of metastatic disease in homozygous MEN2A. Nevertheless, due to the limited number of cases, these findings should be interpreted with caution (16).

The mean diagnostic age for MTC in homozygous patients in the ATA-H and ATA-MOD subgroups was 4.3 and 7.4 years earlier, respectively, than that in their corresponding heterozygous patients. The age difference between the ATA-MOD and ATA-H homozygous groups was approximately 8.7 years; however, none of these differences reached statistical significance (all *P* > 0.05). Therefore, these data may only suggest a weak trend rather than provide evidence of a true biological effect. Previous studies hypothesized that near the RET activation gradient saturation point, the homozygous effects of biallelic mutations in weakly activating variants (e.g., V804) may be more pronounced than those in strongly activating variants (e.g., C634) (13). However, our findings did not support this hypothesis, possibly due to insufficient sample size. The mention of consanguinity, which increases homozygosity in offspring (such as FAII-7), elevates the risk of genetic disorders and may increase the number of affected individuals (e.g., FAIII-3) within the family. However, regional genetic aggregation and founder effects should be taken into account when interpreting the occurrence of homozygous mutations, as they may increase the likelihood of such variants within certain populations (16, 22). Additionally, it is important to note that the carriers in this study were primarily identified due to relatives with MTC, and the observed penetrance may not be applicable to individuals with the same *RET* mutation identified incidentally in the general population. We currently lack sufficient data to analyze sex differences in penetrance among homozygous individuals on a population-wide scale.

Compared to heterozygous patients, homozygous MEN2A cases showed a non-significantly elevated PHEO incidence (27.8% vs. 13.3%, *P* = 0.067). Although the youngest age in homozygous patients was 23 years younger than that in heterozygous patients, the mean diagnostic age for PHEO in homozygous patients was similar to that in heterozygous patients, suggesting that the homozygous state may have a limited impact on the occurrence and progression of PHEO (*P* = 1.000, Mann–Whitney *U* test). Homozygous mutations may result in an increased chance of dimerization between the mutant monomers, thereby partially compensating for the activating effects of the mutant *RET* allele, similar to a “two-hit” mechanism (26).

Additionally, a pair of monozygotic twins, verified through haplotype analyses to carry the MEN2A-harboring germline *RET-*p.C634Y (c.1901G>A) heterozygous mutation, was identified. They reside in the same family and were both 16 years old when their pre-Ctn and pre-CEA levels were found to differ significantly, nearly fourfold. Their postoperative MTC staging was T1aN1bM0 and T2N1bM0, respectively. Despite having similar-sized tumors in their left adrenal glands, they underwent surgery 4 years apart, and their pre-MN levels varied considerably. The twins displayed differing heterogeneity of MEN2A-related MTC and PHEO, indicating the need for individualized management (1–3). Interestingly, their mother had MTC and HPTH but remained free of PHEO at the age of 43, suggesting that her offspring may develop PHEO earlier than she did (22, 27–29).

Previously reported cases of identical twins have included those with MEN2A and MEN2B (20–23). Data from three pairs of identical twins, one with MEN2A and two with MEN2B, revealed inconsistent clinical presentations (e.g., symptomatic or asymptomatic), even among twins of the same age. They also exhibited differential expressivity and tumor-cell heterogeneity of MEN2-related MTC and/or PHEO, such as varying ages of onset and TNM staging. Additionally, they showed different degrees of biochemical levels of Ctn, CEA, and catecholamines/MNs (**Table 3**) (22, 23). These findings suggest that despite the shared genetic background of *RET* mutations, other factors may influence the development and progression of tumors in MEN2 syndrome. A large meta-analysis of twins concluded that such differences are primarily influenced by environmental exposures, developmental variability, and epigenetic factors (30). Further research into the underlying mechanisms is warranted to facilitate personalized precision diagnosis and treatment or monitoring of MEN2.

These findings have direct implications for genetic counseling and clinical management in MEN2A families. Upon identification of a homozygous proband, immediate screening of their parents (*RET* carriers) should be conducted, with suspicion of consanguineous marriage, as such relationships significantly increase the risk of disease in offspring (75% of the proband’s siblings). Preimplantation or prenatal genetic diagnosis may be strongly considered (2, 5–7). For homozygous patients, their offspring will be 100% carriers of the pathogenic mutation. We recommend that, based on *RET* mutation risk stratification and the couple’s preferences, the decision to have children (e.g., ATA-MOD) or not (e.g., ATA-H) should be made. Moreover, the marked phenotypic discordance was observed even in genetically identical twins. Therefore, each twin or any carrier within a family must undergo individualized, risk-adapted surveillance and intervention, rather than assuming concordant disease behavior. This reinforces the need for personalized management strategies based on serial biomarker and imaging assessments, regardless of shared genetics (1, 2, 31).

However, this study has several limitations. First and foremost, the extreme rarity of homozygous *RET* mutations resulted in a small sample size, limiting statistical power to detect significant differences in key outcomes such as age of onset, penetrance, and metastasis risk, particularly in subgroup analyses stratified by ATA risk categories. Second, ascertainment bias likely influenced the findings, as most mutation carriers were identified through symptomatic relatives, potentially overestimating penetrance rates and clinical severity relative to population-level data. Third, the studies included in this review span nearly seven decades, and there may be inherent reporting biases and heterogeneity, which limit the consistency of outcome comparisons. Fourth, inadequate documentation of *RET* testing methodologies and the lack of systematic longitudinal follow-up across studies, compounded by the absence of control groups, impeded robust assessment of treatment outcomes and long-term prognosis. These limitations, inherent to the rarity of the condition and retrospective study designs, underscore the necessity for large-scale multinational collaborations, standardized global registry systems, and integrated molecular analyses to validate these observations.

## Conclusions

Homozygosity for MEN2A accelerates MTC onset, particularly in ATA-MOD mutations, but its impact on PHEO remains unclear. Expressivity and clinical progression can vary even with the same genetic background, and identical twins should also be subject to individual management.

## Data Availability

The datasets presented in this study can be found in online repositories. The names of the repository/repositories and accession number(s) can be found in the article/supplementary material.

## Supplementary material

Supplementary Figure 1 Flowchart of literature search and study selection. Adapted from PRISMA. *From*: Page MJ, McKenzie JE, Bossuyt PM, Boutron I, Hoffmann TC, Mulrow CD, et al. The PRISMA 2020 statement: an updated guideline for reporting systematic reviews. BMJ 2021;372:n71. doi: 10.1136/bmj.n71.

## References

[1] Wells SA Jr Asa SL Dralle H . Revised American Thyroid Association guidelines for the management of medullary thyroid carcinoma. Thyroid. (2015) 25:567–610. doi: 10.1089/thy.2014.0335, PMID: 25810047 PMC4490627

[2] Li SY Ding YQ Si YL . 5P strategies for management of multiple endocrine neoplasia type 2: A paradigm of precision medicine. Front Endocrinol (Lausanne). (2020) 11:543246. doi: 10.3389/fendo.2020.543246, PMID: 33071967 PMC7531599

[3] Machens A Dralle H . Multiple endocrine neoplasia type 2: towards a risk-based approach integrating molecular and biomarker results. Curr Opin Oncol. (2024) 36:1–12. doi: 10.1097/CCO.0000000000001009, PMID: 37975407

[4] Machens A Lorenz K Brandenburg T . The changing face of multiple endocrine neoplasia 2A: from symptom-based to preventative medicine. J Clin Endocrinol Metab. (2023) 108:e734–42. doi: 10.1210/clinem/dgad156, PMID: 36930525

[5] Chen S Li S Zhang J . Preimplantation genetic diagnosis of multiple endocrine neoplasia type 2A using informative markers identified by targeted sequencing. Thyroid. (2018) 28:281–7. doi: 10.1089/thy.2017.0200, PMID: 29378479

[6] Würgler Hansen A Sønderberg Roos LK Løssl K . Preimplantation genetic testing of multiple endocrine neoplasia type 2A. Front Endocrinol (Lausanne). (2020) 11:572151. doi: 10.3389/fendo.2020.572151, PMID: 33178136 PMC7592389

[7] Zhang J Li J Saucier JB . Non-invasive prenatal sequencing for multiple Mendelian monogenic disorders using circulating cell-free fetal DNA. Nat Med. (2019) 25:439–47. doi: 10.1038/s41591-018-0334-x, PMID: 30692697

[8] Huang CN Wu SL Chang TC . RET protooncogene mutations in patients with apparently sporadic medullary thyroid carcinoma. J Formos Med Assoc. (1998) 97:541–6., PMID: 9747064

[9] Lecube A Hernandez C Oriola J Galard R Gémar E Mesa J . V804M RET mutation and familial medullary thyroid carcinoma: report of a large family with expression of the disease only in the homozygous gene carriers. Surgery. (2002) 131:509–14. doi: 10.1067/msy.2002.123006, PMID: 12019403

[10] Elisei R Cosci B Romei C Agate L Piampiani P Miccoli P . Identification of a novel point mutation in the RET gene (Ala883Thr), which is associated with medullary thyroid carcinoma phenotype only in homozygous condition. J Clin Endocrinol Metab. (2004) 89:5823–7. doi: 10.1210/jc.2004-0312, PMID: 15531548

[11] Lesueur F Cebrian A Cranston A Leyland J Faid TM Clements MR . Germline homozygous mutations at codon 804 in the RET protooncogene in medullary thyroid carcinoma/multiple endocrine neoplasia type 2A patients. J Clin Endocrinol Metab. (2005) 90:3454–7. doi: 10.1210/jc.2004-1622, PMID: 15741265

[12] Jaber T Hyde SM Cote GJ Grubbs EG Giles WH Stevens CA . A homozygous RET K666N genotype with an MEN2A phenotype. J Clin Endocrinol Metab. (2018) 103:1269–72. doi: 10.1210/jc.2017-02402, PMID: 29408964

[13] Schirwani S Fraser S Mushtaq T Chengot P Mavrogiannis LA Jewell R . Homozygosity for the pathogenic RET hotspot variant p.Cys634Trp: A consanguineous family with MEN2A. Eur J Med Genet. (2021) 64:104141. doi: 10.1016/j.ejmg.2021.104141, PMID: 33450337

[14] Demir Gündoğan B Sağcan F Tuğ Bozdoğan S Balcı Y Tuncel Daloğlu F Çağlar Çıtak E . Vandetanib in a child affected by neurofibromatosis type 1 and medullary thyroid carcinoma with both NF1 and homozygous RET proto-oncogen germ-line mutations. J Clin Res Pediatr Endocrinol. (2021) 13:342–6. doi: 10.4274/jcrpe.galenos.2020.2020.0051, PMID: 32702947 PMC8388045

[15] Vuylsteke A Hannes L Brems H Devis K Renard M Uyttebroeck A . Germline founder variant c.1998delinsTTCT in the RET oncogene: a cohort study in 15 Belgian families. Eur J Endocrinol. (2023) 189:402–8. doi: 10.1093/ejendo/lvad126, PMID: 37713609

[16] Machens A Dralle H . Accelerated MEN2A in homozygous RET carriers in the context of consanguinity. Eur J Endocrinol. (2024) 190:K43–6. doi: 10.1093/ejendo/lvae025, PMID: 38465999

[17] Brown WJ Barajas L Waisman J De Quattro V . Ultrastructural and biochemical correlates of adrenal and extra-adrenal pheochromocytoma. Cancer. (1972) 29:744–59. doi: 10.1002/1097-0142(197203)29:3<744::aid-cncr2820290331>3.0.co;2-n, PMID: 4334239

[18] Pohl G Boeckl O Galvan G Salis-Samaden R Steiner H Thurner J . Konkordantes medulläres Schilddrüsenkarzinom bei eineiigen Zwillingen mit diskordantem Phäochromozytom (Sipple-Syndrom). Wien Klin Wochenschr. (1977) 89:481–4. 899022

[19] Reza MJ Young RT Van Herle AJ DeQuattro V Cole HS Brown J . Multiple endocrine adenomatosis type II (Sipple’s syndrome) in twins. West J Med. (1975) 123:441–6., PMID: 1199100 PMC1130410

[20] Galera H Gonzalez-Campora R Matilla A Martin I . Multiple endocrine neoplasia type 2b in twins. Histopathology. (1982) 6:111–9. doi: 10.1111/j.1365-2559.1982.tb02706.x, PMID: 7199015

[21] Qi XP Jin BY Li PF Wang S Zhao YH Cao ZL . RET S409Y germline mutation and associated medullary thyroid carcinoma. Thyroid. (2019) 29:1447–56. doi: 10.1089/thy.2018.0385, PMID: 31364476

[22] Qi XP Zhao JQ Fang XD Lian BJ Li F Wang HH . Spectrum of Germline RET variants identified by targeted sequencing and associated Multiple Endocrine Neoplasia type 2 susceptibility in China. BMC Cancer. (2021) 21:369. doi: 10.1186/s12885-021-08116-9, PMID: 33827484 PMC8028819

[23] Rosen JE Lloyd RV Brierley JD Grogan RH Haddad R Hunt JL . Thyroid- medullary (chapter 74). In: AJCC cancer staging manual, 8th ed. Springer International Publishing, New York City, NY (2017). p. 899–909. doi: 10.1007/978-3-319-40618-3_74

[24] Schuchardt A D’Agati V Larsson-Blomberg L Costantini F Pachnis V . Defects in the kidney and enteric nervous system of mice lacking the tyrosine kinase receptor Ret. Nature. (1994) 367:380–3. doi: 10.1038/367380a0, PMID: 8114940

[25] Machens A Gimm O Hinze R Höppner W Boehm BO Dralle H . Genotype-phenotype correlations in hereditary medullary thyroid carcinoma: oncological features and biochemical properties. J Clin Endocrinol Metab. (2001) 86:1104–9. doi: 10.1210/jcem.86.3.7290, PMID: 11238493

[26] Huang SC Koch CA Vortmeyer AO Pack SD Lichtenauer UD Mannan P . Duplication of the mutant RET allele in trisomy 10 or loss of the wild-type allele in multiple endocrine neoplasia type 2-associated pheochromocytomas. Cancer Res. (2000) 60:6223–6. 11103773

[27] Mucha L Leidig-Bruckner G Frank-Raue K Bruckner T Kroiss M Raue F . German study group for rare thyroid cancer. Phaeochromocytoma in multiple endocrine neoplasia type 2: RET codon-specific penetrance and changes in management during the last four decades. Clin Endocrinol (Oxf). (2017) 87:320–6. doi: 10.1111/cen.13386, PMID: 28605116

[28] Machens A Lorenz K Weber F Brandenburg T Führer-Sakel D Dralle H . Genotype-specific development of MEN 2 constituent components in 683 RET carriers. Endocr Relat Cancer. (2024) 31:e240038. doi: 10.1530/ERC-24-0038, PMID: 38753300

[29] Machens A Lorenz K Weber F Dralle H . Sex differences in MEN 2A penetrance and expression according to parental inheritance. Eur J Endocrinol. (2022) 186:469–76. doi: 10.1530/EJE-21-1086, PMID: 35130180

[30] Polderman TJ Benyamin B de Leeuw CA Sullivan PF van Bochoven A Visscher PM . Meta-analysis of the heritability of human traits based on fifty years of twin studies. Nat Genet. (2015) 47:702–9. doi: 10.1038/ng.3285, PMID: 25985137

[31] Kurzawinski TR Butler CR Aziz TA . MEN2: surgical precision in the era of precision medicine. Endocr Relat Cancer. (2025) 32:e240251. doi: 10.1530/ERC-24-0251, PMID: 40340909 PMC12147403

