# Supplementary material for: Rare cases in two Chinese MEN2A families with RET C634Y germline mutation—a homozygous female patient and heterozygous identical twins: a systematic review of literature
Source: Front Endocrinol (Lausanne). 2026 Feb 6;17:1690431. doi: 10.3389/fendo.2026.1690431 (PMC12921576; doi:10.3389/fendo.2026.1690431)
Supplement: Supplementary Figure 1 — Flowchart of literature search and study selection. Adapted from PRISMA. From: Page MJ, McKenzie JE, Bossuyt PM, Boutron I, Hoffmann TC, Mulrow CD, et al. The PRISMA 2020 statement: an updated guideline for reporting systematic reviews. BMJ 2021;372:n71. doi: 10.1136/bmj.n71. [file Image1.pdf]

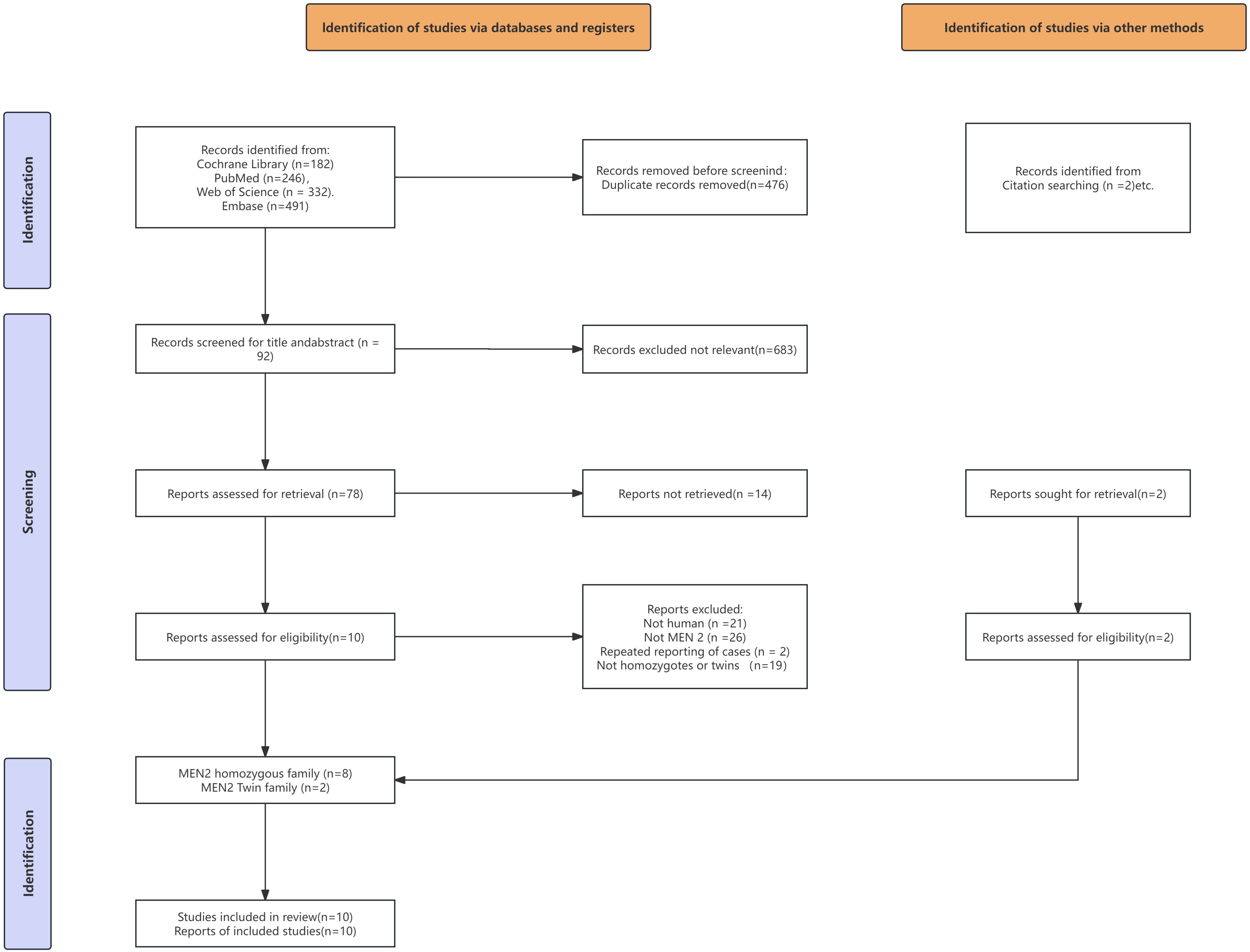
Identification of studies via databases and registers
Identification of studies via other methods
Identification
Records identified from:
Cochrane Library (n=182)
PubMed (n=246),
Web of Science (n = 332).
Embase (n=491)
Records removed before screenind:
Duplicate records removed(n=476)
Records identified from
Citation searching (n =2)etc.
Screening
Records screened for title andabstract (n = 92)
Records excluded not relevant(n=683)
Reports assessed for retrieval (n=78)
Reports not retrieved(n =14)
Reports sought for retrieval(n=2)
Reports assessed for eligibility(n=10)
Reports excluded:
Not human (n =21)
Not MEN 2 (n =26)
Repeated reporting of cases (n = 2)
Not homozygotes or twins (n=19)
Reports assessed for eligibility(n=2)
Identification
MEN2 homozygous family (n=8)
MEN2 Twin family (n=2)
Studies included in review(n=10)
Reports of included studies(n=10)
