# Supplementary material for: Rare cases in two Chinese MEN2A families with RET C634Y germline mutation—a homozygous female patient and heterozygous identical twins: a systematic review of literature
Source: Front Endocrinol (Lausanne). 2026 Feb 6;17:1690431. doi: 10.3389/fendo.2026.1690431 (PMC12921576; doi:10.3389/fendo.2026.1690431)
Supplement: Supplementary file 2 [file Table1.doc]

**Supplementary Table S1.** Presentation of 22 *RET*-ﬂanking microsatellite markers in Twins

| **STR gene locus** | **FBⅢ-1** | **FBⅢ-2** |
| --- | --- | --- |
| D3S1358 | 15, 17 | 15, 17 |
| D13S317 | 8, 12 | 8, 12 |
| D7S820 | 11, 12 | 11, 12 |
| D16S539 | 9, 10 | 9, 10 |
| CSF1PO | 11, 12 | 11, 12 |
| Penta D | 10, 11 | 10, 11 |
| TPOX | 8, 11 | 8, 11 |
| D19S433 | 15.2, 15.2 | 15.2, 15.2 |
| D5S818 | 10, 12 | 10, 12 |
| D21S11 | 29.1, 31.1 | 29.1, 31.1 |
| D2S441 | 11, 12 | 11, 12 |
| Penta E | 5, 14 | 5, 14 |
| Amel | X, Y | X, Y |
| TH01 | 9, 10 | 9, 10 |
| D8S1179 | 11, 12 | 11, 12 |
| D2S1338 | 23, 23 | 23, 23 |
| FGA | 20, 21 | 20, 21 |
| D6S1043 | 14, 19 | 14, 19 |
| D12S391 | 15, 19 | 15, 19 |
| vWA | 17, 18 | 17, 18 |
| D18S51 | 12, 15 | 12, 15 |
| D1S1656 | 15, 16 | 15, 16 |

FB, family B; STR, short tandem repeats
