# Supplementary material for: Rare cases in two Chinese MEN2A families with RET C634Y germline mutation—a homozygous female patient and heterozygous identical twins: a systematic review of literature
Source: Front Endocrinol (Lausanne). 2026 Feb 6;17:1690431. doi: 10.3389/fendo.2026.1690431 (PMC12921576; doi:10.3389/fendo.2026.1690431)
Supplement: Supplementary file 3 [file Table2.doc]

**Supplementary Table S2.** Comparative of MTC and PHEO Characteristics Between Patients with *RET* Homozygous and Heterozygous Mutations in the Same Generation

| **Characteristic** | **Homozygous Mutation (n = 18)** | **Heterozygous Mutation (n = 49)** | ***P*** |
| --- | --- | --- | --- |
| MTC diagnosed, n (%) | 15 (83.3%) | 15 (30.6%) | <0.001 |
| Among MTC patients: N1 metastasis, n (%) | 8/15 (53.3%) | 1/15 (6.7%) | 0.017 |
| PHEO affected, n (%) | 5/18 (27.8%) | 2/15 (13.3%) | 0.067 |
| Mean age at initial PHEO diagnosis, years | 37.75 ± 18.43 [14–59]^#^ | 39.5 ± 3.54 [37–42]^#^ | 1.000* |

MTC, Medullary thyroid carcinoma; PHEO, Pheochromocytoma

^#^Mean ± SD [IQR]; SD, standard deviation; IQR, Inter-Quartile Range

*Mann-Whitney U test
