# Supplementary material for: Rare cases in two Chinese MEN2A families with RET C634Y germline mutation—a homozygous female patient and heterozygous identical twins: a systematic review of literature
Source: Front Endocrinol (Lausanne). 2026 Feb 6;17:1690431. doi: 10.3389/fendo.2026.1690431 (PMC12921576; doi:10.3389/fendo.2026.1690431)
Supplement: Supplementary file 4 [file Table3.doc]

**Supplementary Table S3.** Comparative Analysis of Age at Onset of MTC in *RET* Homozygous and Heterozygous Patients

| **Group** | | **n** | **Mean ± SD** | **Min** | **Max** | ***t*** | **df** | ***P*** |
| --- | --- | --- | --- | --- | --- | --- | --- | --- |
| homozygous carrier | | 15 | 33.40 ± 17.971 | 5 | 59 |  |  |  |
| heterozygous carrier | | 15 | 39.60 ± 12.944 | 14 | 61 |  |  |  |
|  | |  |  |  |  | -1.084 | 28 | 0.144 |
| high-risk | homozygous carrier | 6 | 28.17 ± 20.721 | 5 | 56 |  |  |  |
|  | heterozygous carrier | 6 | 32.50 ± 12.988 | 14 | 46 |  |  |  |
|  |  |  |  |  |  | -0.337 | 10 | 0.337 |
| moderate-risk | homozygous carrier | 9 | 36.89 ± 16.205 | 13 | 59 |  |  |  |
|  | heterozygous carrier | 9 | 44.33 ± 11.169 | 30 | 61 |  |  |  |
|  |  |  |  |  |  | -1.135 | 16 | 0.137 |

SD, standard deviation; IQR, Inter-Quartile Range; Min, Minimum; Max, Maximum; df, degree of freedom.
